# PrestoCell: A persistence-based clustering approach for rapid and robust segmentation of cellular morphology in three-dimensional data

**DOI:** 10.1371/journal.pone.0299006

**Published:** 2024-02-29

**Authors:** Yue Wu, Ingrid Brust-Mascher, Melanie G. Gareau, Jesus A. De Loera, Colin Reardon

**Affiliations:** 1 Department of Computer Science, UC Davis, Davis, California, United States of America; 2 Department of Anatomy, Physiology and Cell Biology, School of Veterinary Medicine, UC Davis, Davis, California, United States of America; 3 Department of Mathematics, UC Davis, Davis, California, United States of America; Universitat Ulm, GERMANY

## Abstract

Light microscopy methods have continued to advance allowing for unprecedented analysis of various cell types in tissues including the brain. Although the functional state of some cell types such as microglia can be determined by morphometric analysis, techniques to perform robust, quick, and accurate measurements have not kept pace with the amount of imaging data that can now be generated. Most of these image segmentation tools are further burdened by an inability to assess structures in three-dimensions. Despite the rise of machine learning techniques, the nature of some biological structures prevents the training of several current day implementations. Here we present PrestoCell, a novel use of persistence-based clustering to segment cells in light microscopy images, as a customized Python-based tool that leverages the free multidimensional image viewer Napari. In evaluating and comparing PrestoCell to several existing tools, including 3DMorph, Omipose, and Imaris, we demonstrate that PrestoCell produces image segmentations that rival these solutions. In particular, our use of cell nuclei information resulted in the ability to correctly segment individual cells that were interacting with one another to increase accuracy. These benefits are in addition to the simplified graphically based user refinement of cell masks that does not require expensive commercial software licenses. We further demonstrate that PrestoCell can complete image segmentation in large samples from light sheet microscopy, allowing quantitative analysis of these large datasets. As an open-source program that leverages freely available visualization software, with minimum computer requirements, we believe that PrestoCell can significantly increase the ability of users without data or computer science expertise to perform complex image analysis.

## Introduction

Neuroimmunology in the central nervous system (CNS) affects a diverse array of biology ranging from traumatic brain injury to autism spectrum disorder, a commonality of these studies is the assessment of resident immune cell activation. Microglia are resident immune cells in the brain that survey CNS tissue for damage, or perturbations to homeostasis due to infection or injury [1, 2]. Under homeostatic conditions, microglia are highly ramified having small cell bodies with multiple processes that are surveying the surrounding space. Activation of microglia by danger-associated or pathogen-associated molecular patterns such as extracellular ATP, or Lipopolysaccharide (LPS) respectively not only induces the expression of pro-inflammatory genes, such as cytokines but also results in a less ramified morphology [3]. This distinct change in morphology following cellular activation has therefore been used to interrogate neuroinflammation in a variety of models and animal species. Although several technologies are capable of ascertaining cell type identity and the gene transcription profile of a single cell [3], dissociative techniques not only result in the loss of spatial information but can also induce activation during isolation [3, 4]. Consequently, light microscopy-based methods such as confocal, two-photon, or light sheet microscopy remain standard methodologies to assess microglial activation within defined tissue structures. Despite this “gold standard” approach, there are several variations used for determining cellular activation based on the amount of ramification. These include widefield microscopy, or confocal image data that is simplified by data reduction with the projection of three-dimensional space down to a two-dimensional representation. While these approaches produce image data of the cells of interest, all three-dimensional data is lost. Typically, the degree of microglial ramification is measured in an indirect manner using Sholl analysis [5, 6]. Although this reductionist approach has been the standard for many years, restricting the analysis to 2D data results in significant data reduction and losses in sensitivity.

While the activation state could be better assessed by image segmentation in three-dimensions, this approach has proven to be technically difficult. Typically, microglia are identified by either indirect immunofluorescence with a primary antibody that detects proteins that are highly expressed by microglia, or by using genetically encoded fluorescent proteins with expression controlled by microglia specific promoters. Image processing routines based on simple thresholding to identify the cell type of interest from the background are usually not sufficient for images of cells within tissues due to different background intensities and an increased potential for noise. Several recent advances have been made including 3Dmorph that seeks to extract information on defined cell types, such as microglia, in three-dimensions [7]. Despite this advance, this process relies on user-determined Otsu thresholding, and decisions on parameters such as maximum cell size and process lengths for each object detected. As a further limitation, these prior works cannot handle large image files that represent large physical regions of a tissue. New technological advancements, including tissue clearing methods such as CLARITY [8] and iDISCO [9], coupled with Light sheet microscopy [10] have further increased these difficulties simply due to the ability to acquire large datasets from an entire intact organ, such as the brain, or small model organisms at sub-cellular resolution. Despite this explosive growth in imaging technology, robust quantitative analysis of increasingly large datasets has lagged. Image analysis of these sample types is difficult if not impossible with these software tools. Although elegant segmentation modalities continue to be developed that leverage machine learning algorithms for the identification of cells, such as Stardist [11] and Cellpose [12], these neural network based tools do not function for non-uniform cell types such as microglia. Segmentation of objects with irregular morphology, such as bacteria, using machine learning algorithms has been implemented in Omnipose [13], although application to other cell types typically requires extensive “training” of a model using expert annotated training data. Commercial analysis software such as Imaris (Oxford Instruments), and Neurolucida (MBF Bioscience) also offer these capabilities, however, in addition to the limitations of generating a training data set, there is often a substantial cost associated with these software licenses. All these issues contribute to an inability to perform robust analysis of non-uniform cell types in physically large regions, whole organs, or organisms from biological samples.

Here, we demonstrate a novel image segmentation approach using persistence homology as part of a workflow to identify and segment microglia as a complex cell type. In brief, persistence homology is a tool in topological data analysis, a field in mathematics that seeks to represent discrete elements in a dataset as a geometric space and to understand the relationships between those elements [14, 15]. Persistence homology identifies the most salient features of a data set by using *filtration*, a technique where a threshold varies from a maximum to a minimum value. Topological features of the dataset “emerge” and “die” depending on the threshold values, with the lifespan being the value of the threshold between the death and birth of a structure. This information is used in persistence homology to find the “persistent” structures in the data set by filtering out structures with short lifespans. Although persistence homology has been used in image segmentation [16, 17], the application of persistence based clustering has been limited [18, 19]. Here we show that this novel approach is capable of identifying complex cell morphologies such as microglia in images representing large physical volumes. Our implementation, that we have named “PrestoCell” can perform segmentation at least as well as existing tools, with the added benefit of allowing the user to easily edit the cell masks. We further demonstrate that as PrestoCell makes use of nuclear markers to identify cells, it can accurately segment cells that are physically interacting. Finally, we show that PrestoCell can assist users in the segmentation of microglia from large volumes of brain tissue acquired by light sheet microscopy. These features and benefits of PrestoCell are available across computer platforms and require no commercial software.

## Materials and methods

### Animals

Samples were obtained from mice originally purchased from The Jackson laboratories. All animals had ad libitum access to food and water, and all procedures were approved by the institutional animal care and use committee at University of California Davis, in accordance with the Guide for Care and Use of Laboratory Animals. Mice were euthanized by CO_2_ asphyxiation followed by cervical dislocation according to American Veterinary Medical Association guidelines.

### Tissue processing

Following euthanasia, trans-cardiac perfusion was performed with ice cold saline, followed by 4% PFA. Brains were carefully dissected and placed in formalin for an additional 24 hours. Samples for confocal microscopy were immersed in 30% sucrose solution as part of a standard cryoprotection regimen. Brains were then placed in molds with optimum cutting temperature (OCT, Fisher Scientific), and rapidly frozen in a dry ice chilled isopentane bath. Blocks were mounted in a cryostat and 40 μm thick coronal tissue sections were obtained.

### Antibody staining

A standard tissue staining protocol was used to label specific cell types. In brief, slides were washed with PBS, and incubated in blocking solution (5% BSA, 5% normal donkey serum) for 1h before incubation with rabbit anti-IBA-1 antibody (catalog number 019–19741; Wako) for 16 h at 4°C, 1:300). After extensive washing donkey anti-rabbit Alexa Fluor 546 was applied for 1h at RT, 1:500 (Invitrogen, cat# A10040), washed extensively and sections were stained with DAPI (1:5000, PBS TritonX100 0.1% v/v). Coverslips were mounted with prolong gold antifade reagent (Invitrogen), allowed to cure and kept at 4°C until imaged.

### Confocal microscopy

Data from samples mounted on slides were acquired with a Leica SP8 STED 3X confocal microscope, equipped with a white light laser (using a 557 nm excitation for Alexa Fluor 546) and a 405 laser (used for DAPI excitation). Images were acquired using a 63x/1.4NA objective, with areas larger than the field of view captured by imaging of multiple overlapping segments (10% overlap), with Z planes acquired at a 0.3 μm step size. Tiles were either processed individually or merged into a larger image before microglia segmentation.

### Light sheet microscopy

After necropsy and perfusion, brains were removed and placed into 4% PFA to ensure proper fixation before tissue clearing using iDISCO(ace) as previously reported [9]. In brief, tissues were pretreated with incubations in (i) 25% acetone, (ii) 50% acetone, (iii) 25% acetone, (iv) PBS, (v) PBS / 30% sucrose at room temperature, followed by incubation in PBS/30% sucrose/1% H_2_O_2_/10 mM EDTA-Na (pH 8.0) at 4°C overnight. Tissues were permeabilized (PBS/0.2% Triton X-100/0.1% deoxycholate/10% DMSO/10 mM EDTA (pH 8.0) overnight), blocked (PBS/0.2% Triton X-100/10% DMSO/5% normal donkey serum at room temperature for two days), and incubated with primary antibody (rabbit anti-IBA-1 1:500 dilution in PBS/0.1% Tween 20/heparin (10 μg/ml)/ 5% normal donkey serum) at 37°C for 4 days. Tissues were washed in PBS/0.1% Tween 20/heparin (10 μg/ml) at 37°C for 48 hours with multiple buffer changes, then incubated in secondary antibody (donkey anti-rabbit Alexa Fluor 546 diluted 1:1000 in PBS/0.1% Tween 20/heparin (10 μg/ml)/ 5% normal donkey serum) at 37°C for 72 hrs. Tissues were washed in PBS/0.1% Tween 20/heparin (10 μg/ml) at 37°C for 48 hours with multiple buffer changes, then incubated in Sytox Deep Red for 24 hrs. After extensive washing, clearing was performed by incubating overnight in each of the following: PBS, 20% methanol twice, 40% methanol, 60% methanol, 80% methanol, and 100% methanol twice. Finally, tissues were incubated in 33% methanol / 67% DCM for 3 hours, 100% DCM twice for 1 hr each, and placed in Ethyl cinnamate (CAS-No: 4192-77-2, EMD Millipore Corporation), before image acquisition with a Zeiss Lightsheet 7 microscope using a 20X immersion objective.

### PrestoCell

#### Theory of persistence-based clustering applied to image data

**Fig 1:**
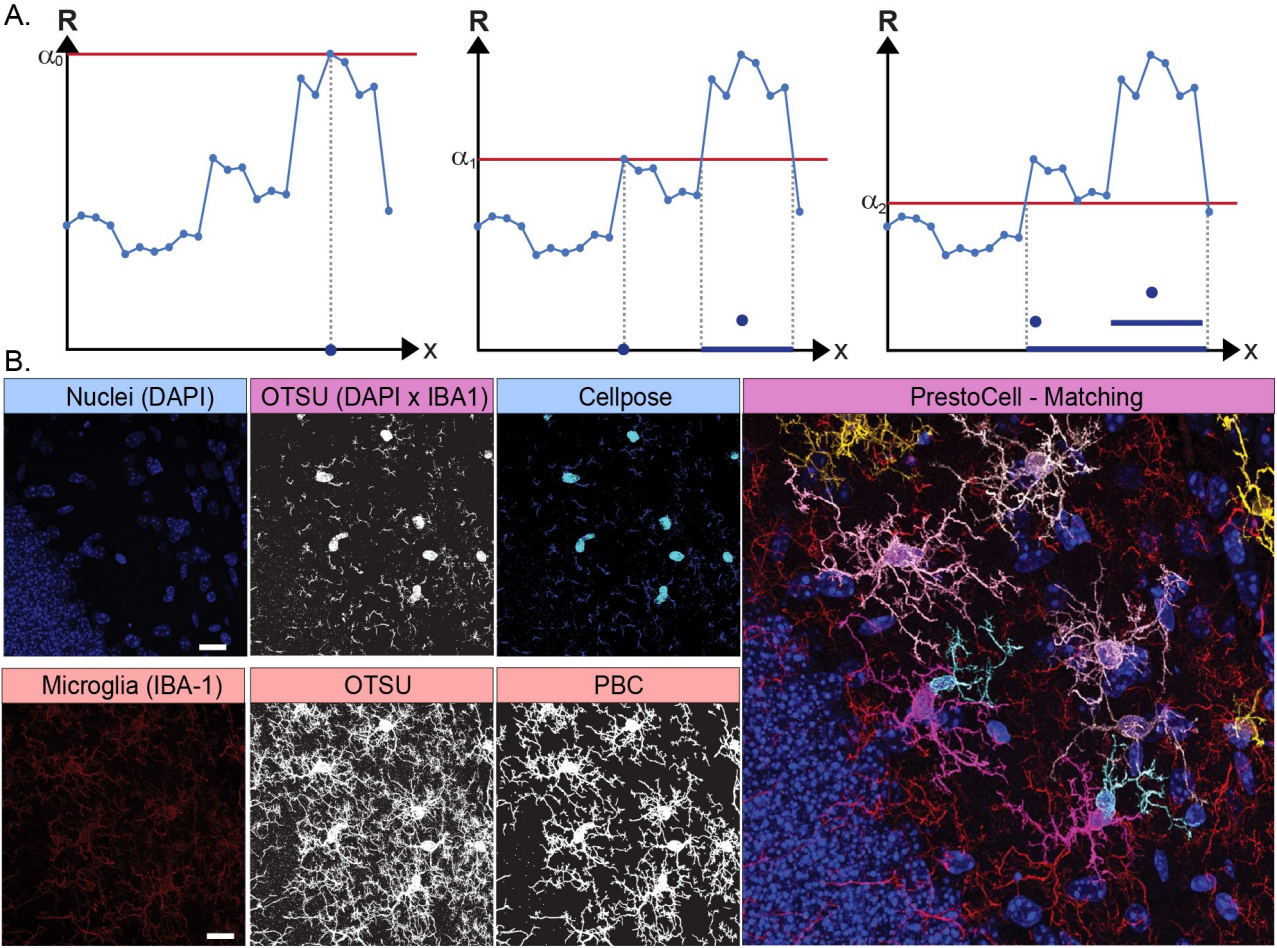
Persistence based clustering and an overview of PrestoCell. **(A)** In a 1D example of PBC, the filtration in PH starts with the threshold α being the maximum value of the data (α_0_) with a single component, then decreasing α to the lowest value. When α is decreased to α_1_, a new component emerges (there are now 2 components). This component dies when α reaches the value α_2_. A persistence threshold δ is then used to decide if a component is an independent cluster or should be merged with another cluster. When looking for potential merges, we only consider adjacent components because all microglia have 1 connected component. **(B)** Overview of the PrestoCell process, where the raw tiff image z-stack is split into channels containing the nuclei (DAPI) and microglia (IBA-1). PrestoCell multiplies the nuclei and microglia channel to identify candidate microglia. Nuclear segmentation is performed using Cellpose and allows the user to refine the predicted nuclear masks. PrestoCell performs PBC on the channel containing the microglia data. The nuclear masks and predicted cells are then matched, and user refinement of the IBA-1 mask can be performed.

Putative microglia are segmented using confocal microscopy data where immunoreactivity for the IBA-1 protein has been detected. Here we perform cell segmentation by using the previously published persistence-based clustering (PBC) software “Tomato” [20], which is a topological segmentation code based on persistence homology. This PBC is derived from Persistence Homology (PH) where a filtration is applied to the input data creating a lifespan of topological features. Prominent structures, with lifespans exceeding a persistence threshold δ, are declared clusters. Structures with lifespans smaller than δ will be potentially merged with adjacent prominent structures. If there are no prominent structures in their immediate neighborhood, they will be considered noise. As an example with 1D data, filtration starts by setting the threshold (α_0_) to the maximum value of the data resulting in a single component **(Fig 1A, left panel)**. The threshold value is then decreased, and at a new value (α_1_), a new component emerges for a total of two components **(Fig 1A, middle panel)**. Further reducing the threshold (α_2_) causes the death of this component **(Fig 1A right panel)**. The persistence threshold δ will determine if components are discrete clusters or should be merged with an adjacent cluster, as each microglia is a group of connected components **(Fig 1A)**. (More details and information about the mathematical foundations and algorithms can be found in [20, 21]).

*Segmentation of confocal images using PrestoCell*. ***Overview*.** PrestoCell performs segmentation on a Tag Image File Format (tiff) file that contains two channels, the first representing nuclei (DAPI), and the second channel corresponding to the cellular marker of interest (IBA-1) **(Fig 1B)**. Once the user has specified the image channel containing microglia PrestoCell performs OTSU thresholding, followed by analysis of this result by persistence-based clustering using Tomato. To segment nuclei that overlap IBA-1, PrestoCell uses Cellpose, a deep neural network trained on very large, annotated data sets, that has superior performance in predicting objects with a uniform morphology [12] **(Fig 1B)**. Since PBC is an unsupervised machine learning method, the nuclear segmentation step allows PrestoCell to refine the microglia segmentation in three discrete ways. First, microglia predicted by PrestoCell that lack nuclei, and therefore are likely incomplete cells, or potential artifacts are removed. Second, clusters from the microglia segmentation that overlap with the same nucleus are merged, as they belong to the same cell. Lastly, clusters that overlap with more than one nucleus are split using a heuristic algorithm we created in PrestoCell to split the cluster into subclusters such that there is a one-on-one correspondence between nuclei and clusters. Clusters can be edited by the user to improve the segmentation **(Fig 1B)**.

**Fig 2:**
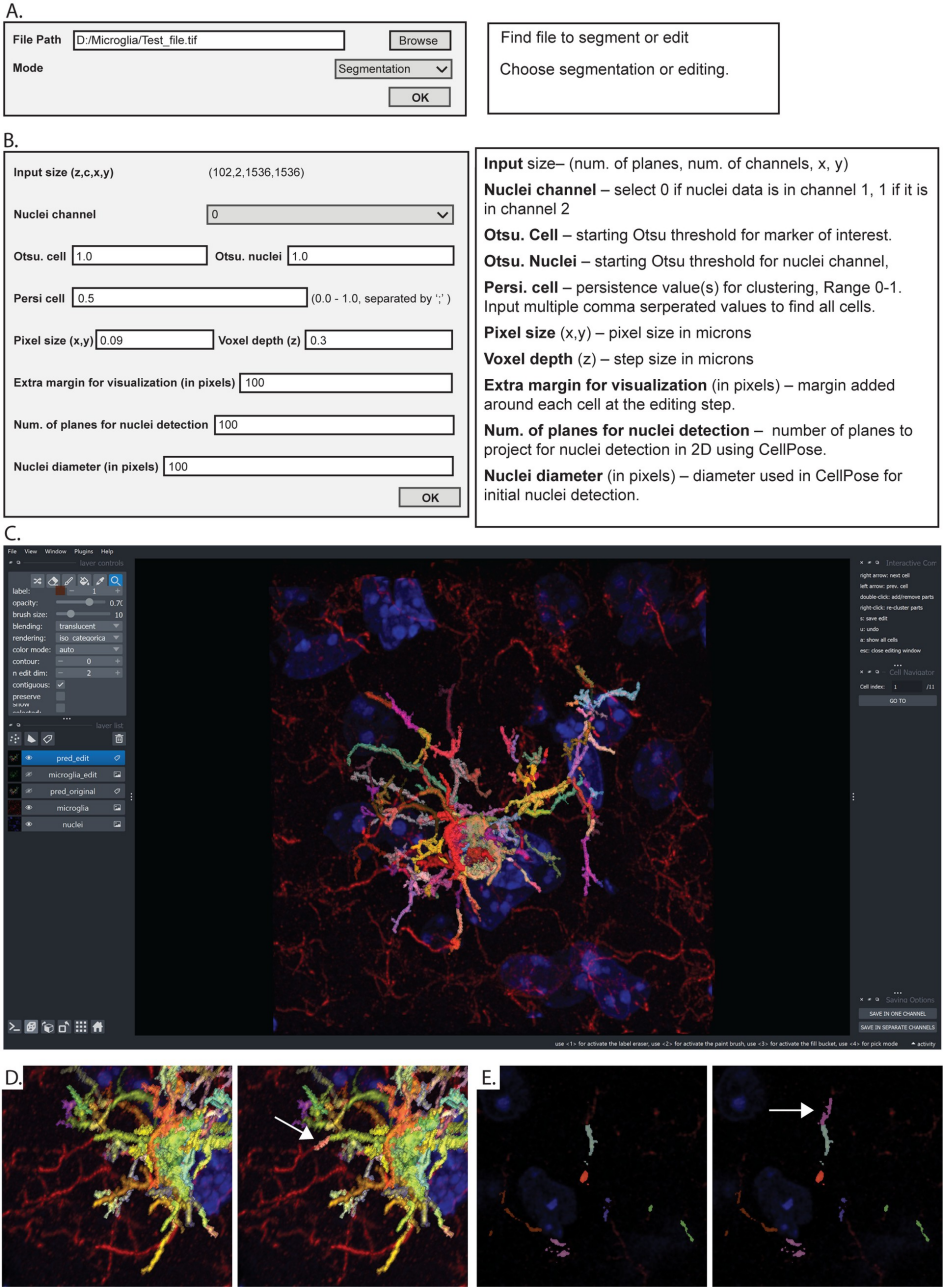
Use of PrestoCell. **(A)** Users interact with PrestoCell through a graphical user interface to select the file they wish to process and the operation (segmentation or editing). **(B)** The user enters additional information about the file and adds parameters for the segmentation routine. **(C)** Editing of the segmentation can be performed using Napari in **(D)** 3D, or **(E)** 2D by using the mouse to add or remove clusters. Here we show that the segmentation mask predicted by PrestoCell is missing elements that can be added to build a segmentation acceptable to the user (arrow).

*Using PrestoCell*. Users begin image segmentation by starting PrestoCell from the command line in a Python environment. A graphical user interface (GUI) directs the user to select the desired file and operation. Here we provide the user the ability to segment an image, or refine a previous segmentation if one exists **(Fig 2A)**. The number of z planes, channels, and dimensions (x and y) of the selected input image are displayed, and the user is asked to provide additional information for segmentation **(Fig 2B)**. PrestoCell leverages the open-source Python-based image viewer Napari, providing the user with an interactive editing feature in two and three-dimensions **(Fig 2C–2E)**. This unique feature allows the user to rotate and modify the segmentation. To allow easy editability, PrestoCell shows each cell with the largest persistence value that produced a putative mask, as the largest possible cell mask. The lowest persistence value is then used to display the identified clusters for editing. Each cluster is assigned a unique color aiding visualization **(Fig 2C–2E)**. This allows the user to visually inspect the PrestoCell suggested mask, refining the segmentation by adding or removing component clusters while viewing the mask and original data in 3D space or 2D planes **(Fig 2D and 2E)**. In the depicted 2D editing window within Napari, the extent of the cell process can be added by clicking the mouse button on the desired adjacent structure **(Fig 2E)**. This is the key to the user-friendly interactive interface with mask refinement at a cluster level, making editing efficient and easy.

#### Comparison to segmentation with other tools

- **Imaris:** Segmentation in Imaris (v10) was performed using “Surface Creation” and selecting LABKIT for the thresholding step. This thresholding uses the machine learning classification kit in FIJI [22], where the user trains a model to distinguish between the foreground and background. These results are returned to Imaris to complete surface creation, where each cell is manually selected, and individual masks are saved as separate surfaces. Cells that are touching are split using the “cut surface” option. Cells requiring multiple splitting events or complex cutting geometries, were not split.
- **3DMorph:** Segmentation of microglia was modified from the original publication [7] using Imaris to aid validation of the segmentation process [23]. A custom modification to the MATLAB script was made to allow the export of unedited 3DMorph masks for all subsequent analysis. User refinement of these masks in Imaris was performed with combining and splitting objects and served as the ground state truth for all comparisons.
- **Omnipose:** Installation with GPU processing enabled was performed as described in the GitHub repository [13]. Segmentation was performed using Jupyter Notebook to generate segmentations with values for “mask_threshold” ranging from [-4 to 4] in increments of 0.5. Values for “flow_threshold” were set to 0.4 as indicated in the documentation for this tool. Cell diameter was adjusted to control the scaling of the image to meet the constraints of the trained model as described. Results of this iteration were saved and evaluated for segmentation with masks evaluated visually and using mathematical tools as described below.

*Evaluation of segmentation quality*. With the rise of machine learning techniques, a plethora of methodologies for the rigorous evaluation of segmentation results have been developed. Although the various analysis modalities seek to compare pixels in a ground state truth image, an annotated image that identifies the structure or feature of interest, each of these measures is subject to different effects of the underlying data set and has unique limitations [24]. With this in mind, we have used multiple comparisons to assess segmentation compared to a ground state truth for each identified cell including Jaccard index (Intersection over Union), F1 score (a.k.a. Dice coefficient, or Sørensen-Dice coefficient) [25], and the percent of false positive and false negative pixels.

## Results

### Image segmentation with PrestoCell

**Fig 3:**
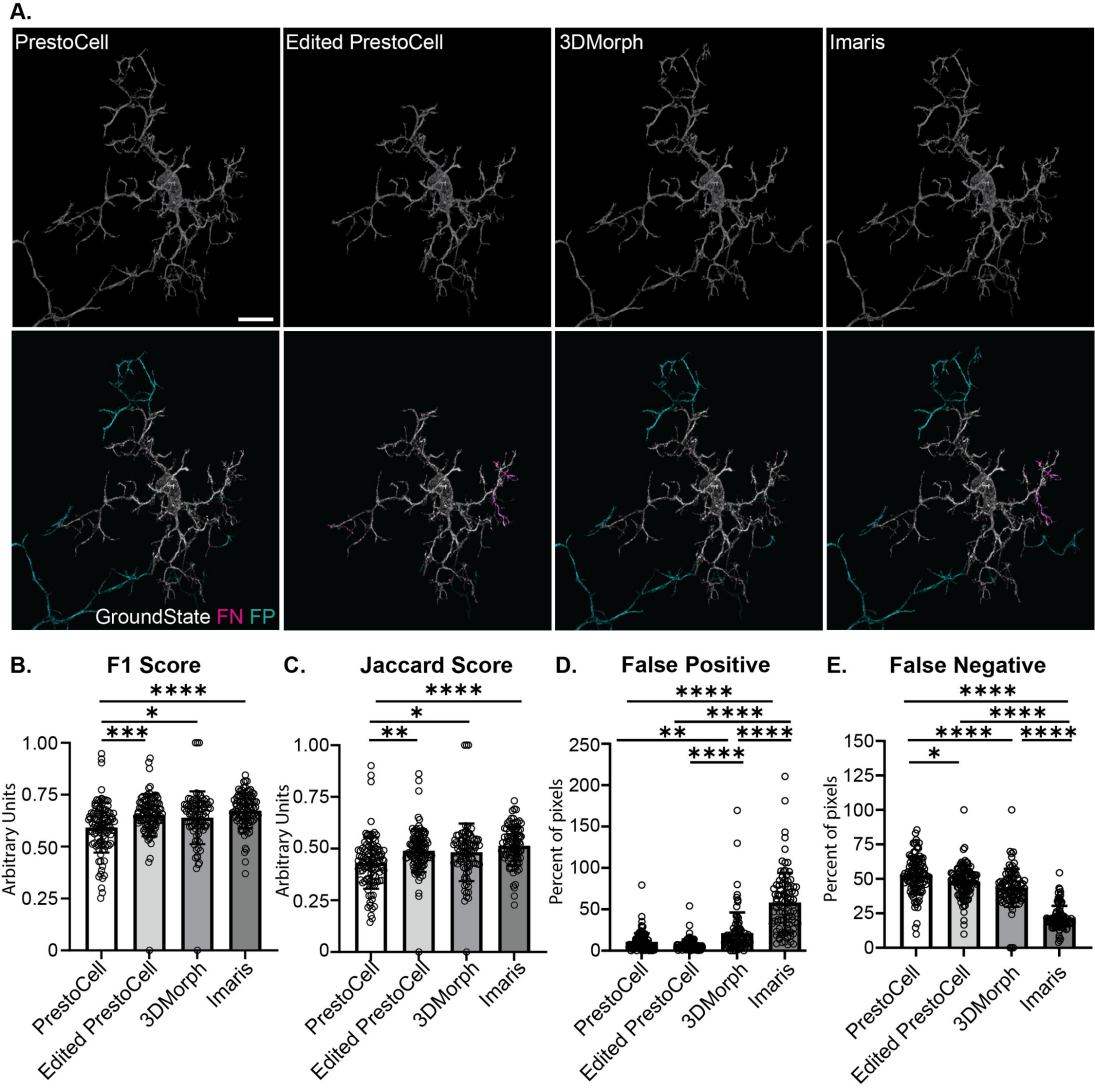
Comparison of PrestoCell segmentation to other tools. **(A)** Segmentation of microglia from light microscopy data produced by PrestoCell, user-edited PrestoCell, 3DMorph, and the LABKIT extension in Imaris (top row). The mask generated by each tool is then compared visually to the ground state (bottom row, white), and image math is performed to identify false positives (“FP”, red) and false negatives (“FN”, cyan). Quantitative analysis comparing each segmentation to the ground state truth was performed using **(B)** F1 score, **(C)** Jaccard score, the **(D)** percent of false positive and **(E)** false negative pixels. Data are presented as mean ± SD, with each segmented cell as a data point. All files are compared to the ground truth, thus the percentage of false positives or negatives can be higher than 100. Scale bar: 10 μm.

PrestoCell was capable of image segmentation and mask generation from 3D confocal images from tiles of confocal Z-stacks with average dimensions of 435 x 435 x 42 μm. Using the workflow described above, we performed image segmentation using PrestoCell **(Fig 3A, top)**. To quantify the accuracy of the segmentation, we compared segmented single cells from the unedited and user-edited PrestoCell outputs to a ground state truth segmentation that was previously prepared **(Fig 3A bottom)**. To determine how PrestoCell compared to other current tools, we conducted segmentations on the same dataset using 3DMorph, and a machine learning-based approach in Imaris **(Fig 3A top)** and compared these to the ground state truth as well **(Fig 3A bottom)**. Visual representation of false positives and false negatives compared to ground state revealed that the PrestoCell outputs without user editing typically have increased false negatives compared to the other tools. Critically, user refinement of the PrestoCell segmentation reduced this type of error. We quantified the performance of each of these tools by comparing the segmentation to the ground state using F1, Jaccard scores, and the false positive and false negative rate for each cell in our data set. As expected from the graphical visualization, unedited PrestoCell segmentations have a lower F1 and Jaccard score, compared to either 3DMorph or Imaris **(Fig 3B and 3C)**. These deficiencies were absent in the user-edited PrestoCell segmentations demonstrating that with simple user feedback, there is no difference in the quality of segmentation produced based on F1 and Jaccard scores. PrestoCell and the edited PrestoCell segmentations had significantly lower false positives compared to the other tools with Imaris producing the most false positives **(Fig 3D)**. False negatives were similar between user-edited PrestoCell segmentations and 3D morph **(Fig 3E)**. These analyses further demonstrated that Imaris platform resulted in significantly less false negatives compared to the other methods.

**Fig 4:**
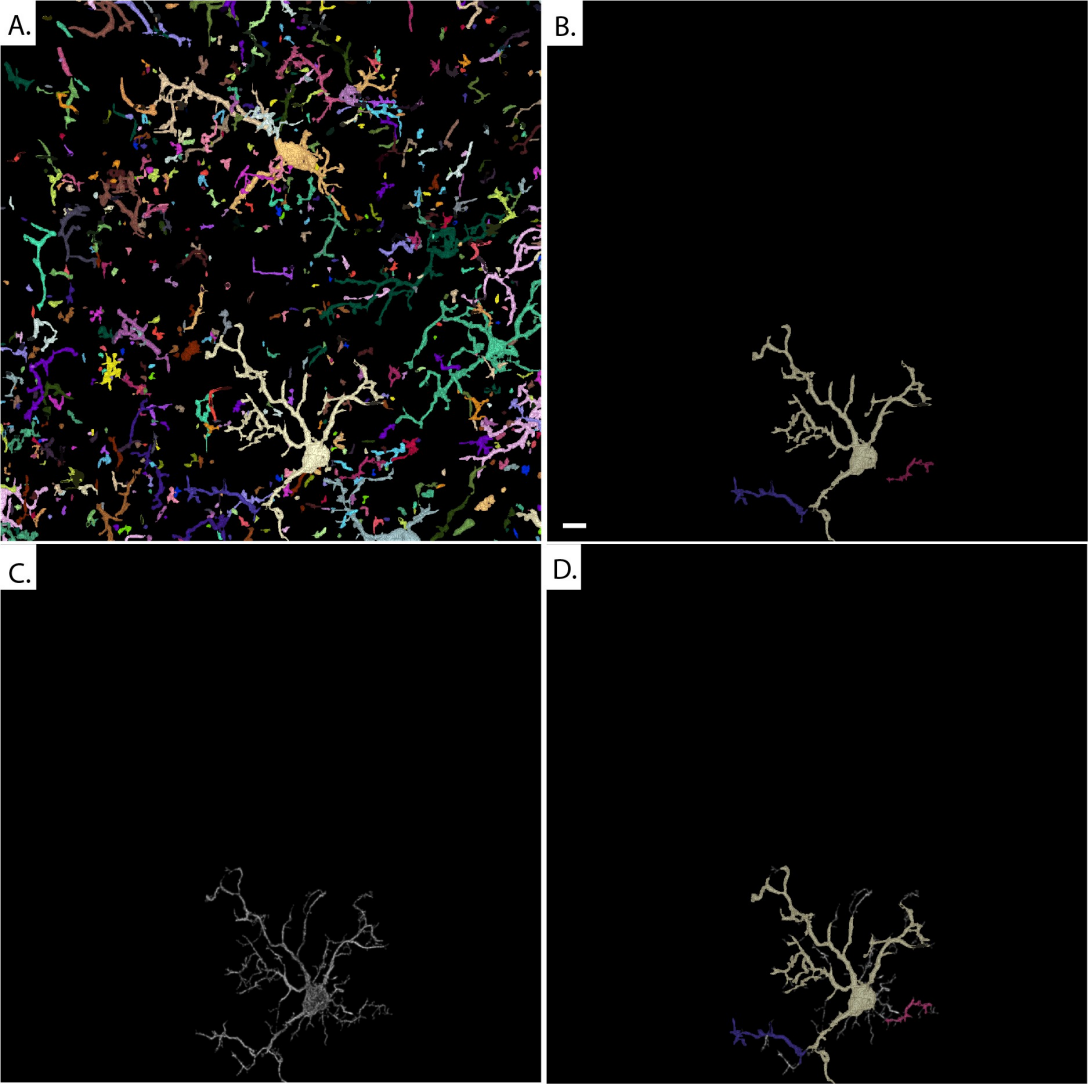
Incomplete segmentation of microglia with Omnipose. **(A)** Using Omnipose on our microglia dataset produced segmentation masks throughout the image. **(B)** In general, these masks were discontinuous and heavily fragmented. Comparison to an individual cell in the **(C)** ground state truth revealed that this cell would be represented by many different **(D)** Omnipose masks. Scale bar: 10 μm.

As a machine learning-based tool, Omnipose has shown a remarkable ability to perform segmentation of light microscopy images of non-uniform cells. Our use of Omnipose was however constrained by an inability to train a custom model on our microglial data due to the sparsity of microglial features in three-dimensions. Segmentation with the pretrained cell models resulted in masks, that were fragments of the individual cells previously identified in our ground state truth **(Fig 4)**. These results suggest that the pre-trained Omnipose model is not capable of segmenting microglia without prior knowledge of the expected result.

Together these data demonstrate that PrestoCell produces image segmentations that are comparable or exceed the results from existing methods. In particular, with the reduced false positives generated from PrestoCell segmentations, PrestoCell will outperform these existing methodologies. In addition, the ease of user editing improves the accuracy of the obtained segmentations.

### PrestoCell-based segmentation of physically interacting cells

**Fig 5:**
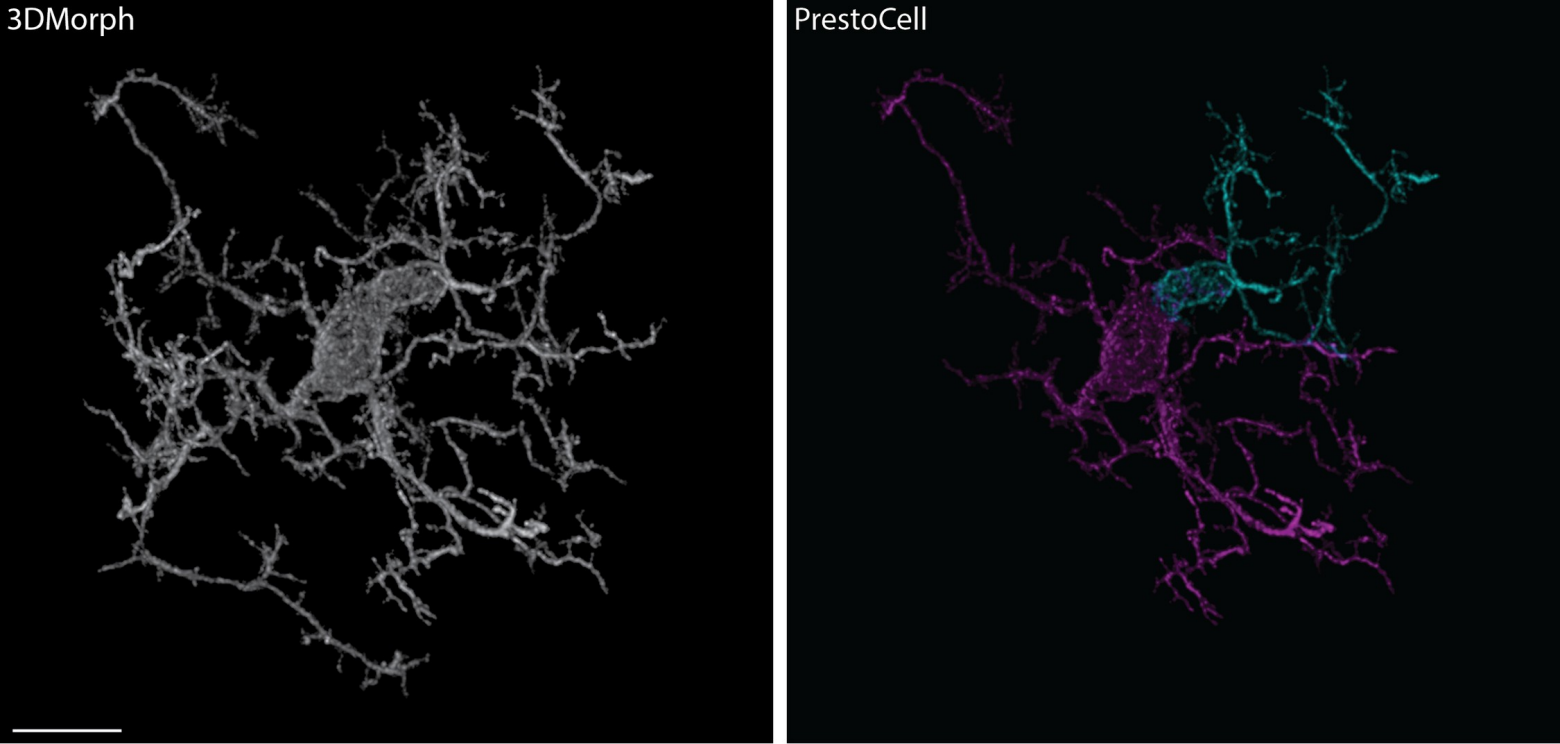
PrestoCell excels in the segmentation of interacting microglia. Interacting microglia were segmented and identified as single cells by 3DMorph (left white). The unedited PrestoCell output demonstrates the power of using nuclear identification, allowing the interacting cells to be resolved (right, pseudo-colored purple, and cyan). Scale bar: 10 μm, representative image of 41 interacting cells identified in 245 discrete masked microglia.

During our evaluation of PrestoCell image segmentation, we noted that PrestoCell was well suited to identify cells in close proximity to one another, or cells that appeared to be physically interacting. These closely juxtaposed cells were not identified as discrete cells in 3DMorph, producing a single segmented cell **(Fig 5)**.

### PrestoCell segmentation of light sheet microscopy data and other cell types

**Fig 6:**
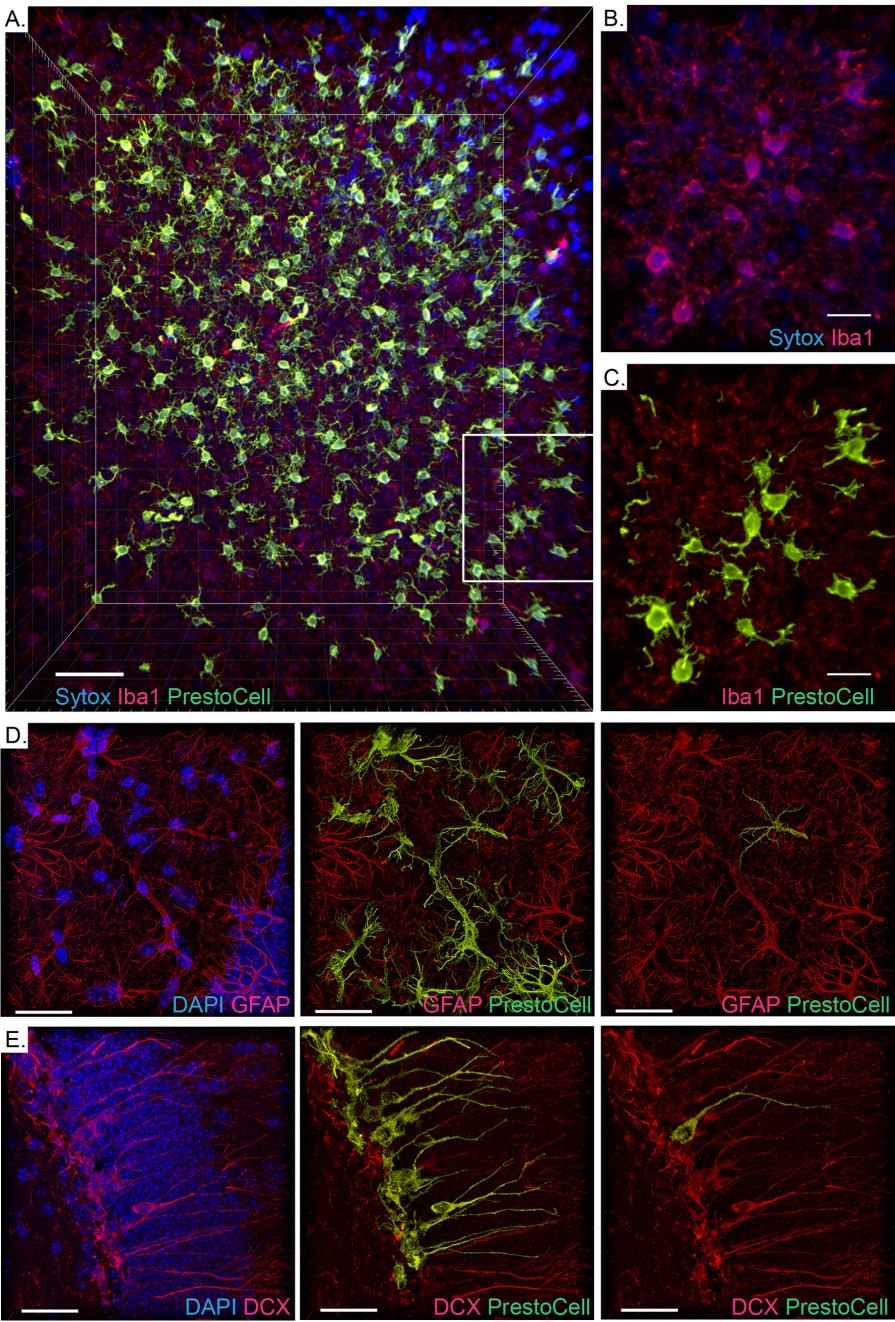
Segmentation of cells within data acquired by confocal and light sheet imaging of the brain. **(A)** An overview of the 664 x 664 x 384 μm of brain volume imaged by light sheet microscopy showing the nuclei (sytox red, blue), microglia (IBA-1, red), and PrestoCell segmentations (green). The indicated subregion shows the concordance between **(B)** DAPI+ microglia and the **(C)** corresponding segmentations. Segmentation of astrocytes (**D,** GFAP+ DAPI+) and newly formed hippocampal neurons (**E,** DCX+ DAPI+), with raw data (left panels), segmentation (middle panels), and a single segmented cell (right panels) shown. Scale bar: 70 μm for **(A)**; 30 μm for **(B-E)**.

Continued development of light imaging modalities capable of sub-cellular resolution throughout a whole organ or even a small model organism has produced remarkable results with few tools to aid quantitative analysis. Here we applied the immunolabeling-enabled three-dimensional imaging of solvent-cleared organs (iDISCO) protocol to the mouse brain allowing optical clearing and immunostaining. PrestoCell was able to segment microglia within a 664 x 664 x 384 μm region (0.169 mm^3^) from a mouse brain subjected to tissue clearing with iDISCO and light sheet microscopy **(Fig 6A–6C)**. This is the largest tested dataset we have attempted to segment using PrestoCell. Attempting to generate segmentations using 3DMorph resulted in the software becoming rapidly resource-constrained and yielded no segmented cells. In addition, PrestoCell is not restricted to microglia segmentation, with masks produced from astrocytes (GFAP+ DAPI+, **Fig 6D**) and newly formed hippocampal neurons (DCX+ DAPI+ **Fig 6E**), allowing isolation of individual cells (**Fig 6D and 6E right panels**). Together these results suggest that the benefits of PrestoCell could be applied to a diverse range of cell types and imaging applications.

## Discussion

Rapid advances in light microscopy technologies have brought about an unprecedented increase in the amount of data generated in the life sciences. Despite the development of techniques to acquire data from entire intact organs including the brain, the ability to assess these data rapidly and quantitatively has been limited by a lack of tools, or approaches that are time-intensive. Traditionally, this analysis has been performed on 2D datasets or maximum intensity projections of 3D data to generate 2D datasets. These approaches result in the loss of data, and therefore the ability to detect small and potentially meaningful biological effects. Better representations of the complexities of cells in 3D can also be achieved by computerized tracing of the raw data. Perhaps unsurprisingly this approach must be completed with great care, is laborious and painstaking when dealing with many individual cells and many biological replicates. While many methodologies and segmentation techniques have been developed for microscopy, few can provide segmentation of irregularly shaped cell types that can be closely juxtaposed. Recent tools such as Cellpose [12], or histopathology samples with QuPath [26] generally do not perform well for analysis of non-uniform cells such as microglia. Other approaches include 3DMorph, a MATLAB-based script designed for the segmentation and analysis of microglia in 3D datasets [7]. Although 3DMorph offers substantial improvements in the segmentation of these cells under specific conditions, there are clear limitations and design choices that can preclude generalized use. Our experience with these prior tools led us to create PrestoCell allowing biologists to segment non-uniform microglia rapidly and accurately. Here we document that cell masks generated by PrestoCell and user-refined PrestoCell masks are equivalent to, or exceed the accuracy of 3DMorph and even a commercial machine learning approach. We further demonstrate that although the ability of 3DMorph to segment cells without requiring a nuclear marker, such as DAPI, is touted as a benefit, there are instances where this choice leads to inaccuracies. The requirement of nuclei for PrestoCell to segment cells provides the ability to confidently segment microglia that are closely juxtaposed or interacting in 3D. In stark contrast, 3DMorph typically groups microglia with these characteristics together. The resulting segmentation mask remains less than the user defined maximum physical size but with two discrete nuclei. While it is possible to separate these cells post-segmentation, the output of cell masks from 3DMorph was not implemented in the original script, preventing users not familiar with MATLAB from achieving this easily. In addition, 3D visualization software with the capacity to edit, such as Imaris, is required to separate aberrant microglia masks. We have previously used this approach [23], although identification of where segmentations should be split or joined in 3D is entirely up to the user, without any guidance to indicate points or regions where two cells are interacting. This requirement of PrestoCell for a secondary marker to identify nuclei is therefore both an advantage, but could be a limitation depending on the nature of the experiment and images acquired. For example, this current implementation of PrestoCell would not be able to function on any samples lacking nuclear co-staining. While an obvious solution is simply to co-stain with DAPI or similar reagents to reveal nuclei, such a requirement may not be possible in certain live cell or intravital microscopy studies. Our results further demonstrate that PrestoCell can segment cell types other than microglia, including astrocytes and neurons. Although we did not perform quantitative analysis of this segmentation, these data are encouraging suggesting that PrestoCell could be used in a wide range of image processing workflows.

PrestoCell has been designed to allow visualization and refinement during the mask generation process. To achieve this, we have leveraged existing tools in the Python language, with visualization and editing performed in 3D using the user-friendly multidimensional Napari viewer. The use of Python and the various tools was purposefully selected to ensure a broad multiplatform use, that does not require a MATLAB license or purchase of expensive visualization software such as Imaris. During the mask refinement process we further specifically highlight “critical points” allowing a user to review and determine if those regions should be included in the final mask, that are based on the PBC outcomes. This reduces the subjectivity of splitting or adding during mask refinement, and is a clear benefit of our approach in PrestoCell. Another clear benefit of PrestoCell over current tools is the ability to perform segmentation on large datasets where 3DMorph simply fails to load the data. These datasets include not only standard confocal image tiles but also the ability to perform segmentation from large pieces of tissue acquired by light sheet imaging. This attribute will allow PrestoCell to aid users in performing quantitative analysis on large volumes of data obtained with new technologies.

Analysis tools have recently been developed to make use of machine-learning approaches. In general, these machine learning algorithms use statistical based models trained on datasets containing the cell type of interest. Training and model development to reduce the potential for bias can be difficult to achieve in practice, and non-commercial software may require a significant knowledge base in data science or machine learning. Given the interest and potential power of these approaches, many commercial image analysis platforms are offering fully integrated solutions or the ability to use open-source tools. These include the use of the LABKIT Fiji plugin for machine learning pixel classification as an extension for Imaris. Segmentations produced using this LABKIT extension generated robust F1 and Jaccard scores. Despite this performance, the number of false positives was significantly increased compared to PrestoCell and 3DMorph. These results suggest that PrestoCell performs as well, or better under select conditions compared to this Imaris extension. We believe that future iterations of PrestoCell or the use of PBC coupled with machine learning could present a powerful next step to improve upon the image segmentation.

Our results to date demonstrate that as a novel use of persistence-based clustering, PrestoCell can provide users across different operating systems with a robust segmentation tool that is easy to use and able to handle large datasets. These benefits of PrestoCell are due to an inherent ability to keep track of and analyze complicated multi-dimensional structures that is derived from our use of mathematical concepts of homology, compared to the relatively simple connectivity-based methods. Critically this implementation is accurate, and in some cases outperforms existing segmentation tools, and will allow users of varying skill levels to begin quantitative analysis of non-uniform cell types including microglia.
